# Epistemic injustice in climate health research

**DOI:** 10.2471/BLT.25.294374

**Published:** 2026-09-01

**Authors:** Verina Wild, Anthony Capon, Jan-Christoph Heilinger, Cristian Timmermann, Mujahid Torwali, Jakelin Troy

**Affiliations:** a Institute for Ethics and History of Health in Society (IEHHS), Medical Faculty, University of Augsburg, Universitätsstr. 2, 86159 Augsburg, Germany.; b Monash Sustainable Development Institute, Monash University, Melbourne, Australia.; c Department of Philosophy, Politics and Economy, Witten/Herdecke University, Witten, Germany.; d Faculty of Arts and Social Sciences, University of Sydney, Sydney, Australia.

In health research, policy and practice, epistemic injustices occur in the production and dissemination of knowledge.^1^^–^^3^ Epistemic injustices involve the unfair treatment of individuals or communities in their capacity as knowers, that is, when they are excluded, discredited, misrepresented or misunderstood in processes of knowledge production, validation and exchange.^4^ The harmful effects of these injustices are well documented, for instance in gender medicine, health research from low- and middle-income countries or in relation to traditional medicine.^5^^,^^6^

The concept of epistemic injustice is useful in understanding the structural origin and extent of imbalances in knowledge production. According to philosopher Miranda Fricker, two main domains of epistemic injustice exist.^4^ Systematic testimonial injustice occurs when a society wrongs or oppresses certain groups by giving them less credibility. If contributions from those groups are not regarded as valid and truthful, they are not included in common knowledge systems, and no action follows.^4^ Systematic hermeneutical injustice occurs when groups experiencing marginalization are disadvantaged in making sense of and communicating their experiences because they have been excluded from shaping collective interpretive resources. This exclusion prevents their particular experiences and insights from being adequately articulated and understood, and their knowledge from becoming intelligible and recognized within dominant knowledge systems.^4^

Here we focus on epistemic injustice in climate health research because of its moral relevance: environmental changes put many people and ecosystems at high risk of harms. All knowledges capable of informing efforts to mitigate the accelerating deterioration of the climate and ecosystems ought to be recognized and taken seriously.

We focus specifically on the underlying conceptual understanding of health because it influences what is researched and by whom, how research is conducted, who is included, and how data and evidence are interpreted.^1^^,^^2^ Value assumptions about what counts influence which projects are funded, published and cited, thereby shaping health policy and practice nationally and globally, ultimately affecting human and planetary well-being.

Our main claim is that climate health research, particularly with its ongoing engagement with Indigenous scholarship,^2^ contributes to rectifying historic epistemic injustices while increasing cognitive diversity. Health researchers and bioethicists ought to contribute collectively to interrogating and ultimately overcoming epistemic injustices.^7^

## Climate health research

Climate change creates risks for health and health equity. Substantial differences exist in the experiences, attitudes and knowledges about what constitutes health risks and how to address them. Climate health research is essential for assessing health impacts and their distribution, and for developing adaptation and mitigation strategies.

In our view, climate health research occupies a critical space because the research field risks perpetuating epistemic injustices, when dominant paradigms still privilege narrow, biomedical or anthropocentric conceptions of health. At the same time, the field is uniquely positioned to advance a richer normative understanding of health as interdependent, considering people and planet, with potentially transformative effects on health research.

Epistemic injustices shape all fields of health research by limiting which normative visions of health are recognized as credible or valuable. Groups who contribute least to climate change, including Indigenous communities, remain underrepresented in health research while being exposed to new health threats, including harvest losses and scarcity of plants for traditional medicines.^6^ Excluding their knowledges and understandings of the world, and failing to recognize the scientific merit of their experiences adapting and responding to climate-related health hazards is unjust and inefficient.^8^

At the same time, global collaboration in climate health research is creating opportunities for plural and more inclusive normative understandings of health, particularly regarding Indigenous knowledges.^9^ Recognizing relational, ecological and collective forms of knowledge reduces injustice and broadens the conceptual and methodological scope of climate health research by integrating perspectives that have long been neglected.^10^ Researchers from all fields of health research and bioethicists ought to recognize this diversity by explicitly questioning value assumptions underlying their work and by addressing epistemic injustices that constrain the moral and conceptual horizons of health research.

## Normative understandings of health

From the perspective of epistemic justice, only conceptions of health that are both theoretically and practically inclusive of diverse ways of knowing result in an adequate normative understanding of health. Such understanding would allow addressing as many needs and interests as possible. Identifying and addressing epistemic injustices is thus an essential part of the ethical contribution to transforming health research. Otherwise, existing intersectional inequities will continue to grant some groups disproportionate epistemic power to define what counts as relevant for health research, set research agendas and determine criteria of success in research. For example, rural Indigenous women often have fewer opportunities to influence scientific, ecological or health-related agendas than other social groups.^11^

Ethically, such power imbalances ought to be identified and corrected to democratize health research agenda-setting and make sure research priorities are set fairly and reflect existing health needs, rather than being dominated by, for example, narrow conceptions of health, commercial interests or entrenched academic incentives.^12^

Indigenous Peoples in diverse settings hold deep, time-tested, holistic understandings of the natural environment and health and are fully aware that the connection to land and waters sustains communities in all aspects of life. In philosophies of health that do not centre on or privilege humans, health is inseparable from the well-being of Place, Land and Country.^9^ *Yarnhealth: Girinyalanha Waluwin*, for example, communicates ideas that Aboriginal people on Wiradjuri land in Australia have shared about health. Such understandings are intimately tied to a conception of health as a deeply interdependent engagement between humans and the environment in which humans contemplate themselves as part of the whole. Recognizing Indigenous knowledges and other interdependent conceptions of health is key to better understanding the relationship between health and environment. These approaches raise awareness of the shortcomings of understandings of health that permit individuals to regard themselves as healthy or well even while community members are suffering or the environment is damaged. Holistic and interdependent understandings of health measure success as collective resilience and thriving, and not simply as individual or isolated achievements. Such normative understandings of health emphasize that everyone’s well-being is ultimately dependent on the ability to collectively live in harmony with the surrounding nature.^13^

Anthropocentric normative conceptions of health must make this interdependence visible in a meaningful and constructive way. This shift includes moving beyond dichotomies such as individual versus population health, present versus future generations or humans versus environment. Indigenous knowledges can offer important insights and correctives towards better integration. To advance a more interdependent understanding of health, respectful dialogue and mutual learning between various theories of health and knowledge systems are essential. While such engagement has started to permeate climate health research, it should be viewed as a collective moral imperative for reaching epistemic justice. This dialogue also carries the potential to enrich bioethical and health research in general, including its concepts, methods and outcomes.

## Doing epistemic justice

Advancing epistemic justice in knowledge production and reducing structural health vulnerabilities is challenging due to deeply ingrained structural injustices and insufficient motivation to change them.^9^ Structural injustice means that social processes and institutional arrangements such as ongoing and new forms of colonialism, exploitation and political marginalization systematically disadvantage some groups while enabling others to benefit.^14^ Advancing epistemic justice in knowledge production therefore demands proactively including historically marginalized voices, recognizing them as trusted knowers, and to understand, expose and address structural knowledge-related injustices. An important element of structural and epistemic justice is to identify where Indigenous knowledges might be instrumentalized or exploited, instead of taking part in respectful co-learning.^15^

Doing epistemic justice is not a matter of taking one local experience and applying, appropriating, normalizing or upscaling Indigenous knowledges across the planet. Each local system of Indigenous knowledges is unique. However, mutual learning across local knowledges and finding collective solutions for all life on the planet is possible. What we advocate for is that local knowledges be understood in their local context, and with this understanding they become part of possible solutions to problems we face on a planetary scale.

The respectful and collaborative dialogue between empirical and normative disciplines, between biomedical and more holistic perspectives, and between Indigenous and other approaches that have different histories and paradigms could be challenging. Mutual understanding and exchange require respect, time and space to produce theoretically sound, culturally sensitive and practicable results.^2^ Here too, the global research community can profit from Indigenous knowledges and practices. Building on knowledges, shared through the practice of Dadirri for example, has been suggested as a possible way to inform and transform conventional scholarly research.^7^ Dadirri, from Yolngu language and cultural practice in north-east Arnhem, Australia, means deep listening, and includes witnessing, hearing and reflecting. Dadirri creates relationships, reciprocity, respect, trust and empathy, and strengthens awareness of one’s surroundings. Performed as a collective, participatory method, it can reveal structures and experiences of oppression in relation to health, well-being and climate.^13^ Applied in climate health research, Dadirri can reduce power imbalances between researchers and participants, and increase agency. Allowing spontaneous contemplations and interactions^13^ might break with standardized research protocols but can align with more justice-oriented approaches such as community-based participatory research.^16^ This approach can reveal hidden health concerns and identify proven and novel adaptive strategies that improve the human-environment relation. Potential value conflicts such as competing interests, notions of stewardship, health–health trade-offs, use of scarce resources and obligations should be discussed in transparent and inclusive ways. Increasing justice and holistic understandings in the underlying concepts of health can also help to set suitable priorities in health research agenda-setting and funding criteria.

To develop a normative concept of health that is beneficial and just for all, addressing structural and epistemic injustices in knowledge production is essential. Climate health research is at risk of repeating the exclusion of historically marginalized voices, resulting in moral harms for all life on the planet. Yet, especially in collaborative responses to epistemic injustices, climate health research has the potential to transform normative understandings of health towards more interdependent approaches and thus to renew health research, policy and practice.
